# Biological treatment evaluation in thermoradiotherapy: application in cervical cancer patients

**DOI:** 10.1007/s00066-023-02185-4

**Published:** 2024-01-04

**Authors:** H. P. Kok, T. D. Herrera, J. Crezee

**Affiliations:** 1 Dept. Radiation Oncology, Amsterdam UMC location University of Amsterdam, Meibergdreef 9, 1105 AZ Amsterdam, The Netherlands; 2 Treatment and quality of life, Cancer biology and immunology, Cancer Center Amsterdam, Amsterdam, The Netherlands

**Keywords:** Hyperthermia, RF heating, Treatment logistics, Biological modelling, Equivalent dose

## Abstract

**Background:**

Hyperthermia treatment quality is usually evaluated by thermal (dose) parameters, though hyperthermic radiosensitization effects are also influenced by the time interval between the two modalities. This work applies biological modelling for clinical treatment evaluation of cervical cancer patients treated with radiotherapy plus hyperthermia by calculating the equivalent radiation dose (EQD_RT_, i.e., the dose needed for the same effect with radiation alone). Subsequent analyses evaluate the impact of logistics.

**Methods:**

Biological treatment evaluation was performed for 58 patients treated with 23–28 fractions of 1.8–2 Gy plus 4–5 weekly hyperthermia sessions. Measured temperatures (T50) and recorded time intervals between the radiotherapy and hyperthermia sessions were used to calculate the EQD_RT_ using an extended linear quadratic (LQ) model with hyperthermic LQ parameters based on extensive experimental data. Next, the impact of a 30-min time interval (optimized logistics) as well as a 4‑h time interval (suboptimal logistics) was evaluated.

**Results:**

Median average measured T50 and recorded time intervals were 41.2 °C (range 39.7–42.5 °C) and 79 min (range 34–125 min), respectively, resulting in a median total dose enhancement (D50) of 5.5 Gy (interquartile range [IQR] 4.0–6.6 Gy). For 30-min time intervals, the enhancement would increase by ~30% to 7.1 Gy (IQR 5.5–8.1 Gy; *p* < 0.001). In case of 4‑h time intervals, an ~ 40% decrease in dose enhancement could be expected: 3.2 Gy (IQR 2.3–3.8 Gy; *p* < 0.001). Normal tissue enhancement was negligible (< 0.3 Gy), even for short time intervals.

**Conclusion:**

Biological treatment evaluation is a useful addition to standard thermal (dose) evaluation of hyperthermia treatments. Optimizing logistics to shorten time intervals seems worthwhile to improve treatment efficacy.

## Introduction

Cervical cancer is the fourth most common cancer in women [1]. Standard treatment of locally advanced tumors is chemoradiation [2], but patients with a medical contraindication to cisplatin-based chemotherapy should be treated with proven effective alternative strategies, e.g., thermoradiotherapy (i.e., radiotherapy combined with hyperthermia). Hyperthermia typically uses a locoregional heating device consisting of a ring of radiofrequency or microwave antennas positioned around the pelvis to increase tumor temperatures to 39–44 °C for 1 h, thereby enhancing radiosensitivity [3]. During the fractionated radiation schedule, hyperthermia is applied once or twice weekly, shortly before or after a radiotherapy fraction. The effectiveness of hyperthermia was demonstrated in randomized phase III trials [4–6]. The Dutch Deep Hyperthermia Trial showed a significant improvement for both 12-year local control (from 37 to 56%, *p* = 0.01) and survival (from 20 to 37%, *p* = 0.03) for thermoradiotherapy compared to radiation alone [7]. No significant differences in late toxicity were observed, indicating the tumor selectiveness of hyperthermia. The RADCHOC study suggested comparable outcomes of chemoradiation and thermoradiation, although the trial was closed prematurely because of low accrual [5].

Hyperthermia thus significantly enhances the effectiveness of radiotherapy, but clinical data show a strong thermal dose–effect relationship [8, 9]. Hyperthermia delivery therefore aims at high tumor temperatures up to 42–43 °C, though patient tolerance is often a limiting factor. The applied power is often limited by a need to prevent excessive normal tissue temperatures, which should remain below 44–45 °C to avoid pain sensations (hotspots) and thermal damage [10]. Therefore, treatment quality is continuously monitored by thermometry probes in the pelvic body cavities, in combination with patient feedback about normal tissue hotspots. When necessary, device settings are adjusted to reduce hotspot temperatures or improve target heating. Standard quality metrics used are indexed temperatures T10, T50, and T90, i.e., the temperatures at least achieved in 10, 50, and 90% of the measurement points during treatment, respectively [11, 12], and the number of equivalent minutes at 43 °C (CEM43) [13]. Despite effective steering protocols and treatment planning strategies to optimize treatment delivery [14–16], treatment-limiting normal tissue temperatures frequently occur due to strong variations in power absorption and perfusion in different tissues.

Although achieved temperatures are a very important factor determining treatment quality, the complex synergy between radiotherapy and hyperthermia means that the effectiveness of hyperthermia is also influenced by parameters other than temperature. An important parameter is the time interval between the radiotherapy fraction and hyperthermia delivery [17, 18]. The therapeutic effectiveness of hyperthermia decreases with increasing time interval. Therefore, additional comprehensive quality metrics that account for temperature and other factors would be useful to predict, evaluate, and optimize clinical results of thermoradiotherapy.

Biological modelling could be very helpful to model dynamic processes responsible for cellular response to treatment, thereby providing more insight into clinical implications of the complex synergy between radiotherapy and hyperthermia [19]. In radiotherapy, biological modelling is quite common to compare fractionation schemes, or to predict tumor control and normal tissue toxicity [20, 21]. These models are usually based on the linear quadratic (LQ) model [22] to account for the radiosensitivity of tumors and normal tissue. Since hyperthermia enhances radiosensitivity, an extended LQ model can be used for biological modelling for thermoradiotherapy [23–26]. Using such a model, the effect of hyperthermia can be translated into an equivalent radiation dose (EQD_RT_), i.e., the dose that would yield the same effect with radiation alone.

Previous studies applied biological modelling for thermoradiotherapy using simulated temperature distributions to obtain more insight into the impact of different treatment parameters or to explore the feasibility of hyperthermia for non-standard indications [24, 27–31]. So far, biological modelling has not been used for evaluation of actual clinical treatments. In this study, we apply biological modelling to evaluate treatment quality in terms of dose enhancement in a cohort of 58 cervical cancer patients treated with thermoradiotherapy. Real treatment data, i.e., clinically measured temperatures and registered time intervals, will be used to estimate the EQD_RT_ in these patients. Additionally, the impact of logistics on the expected EQD_RT_ is evaluated and biological treatment evaluation for individual patients is illustrated for two treatment sessions.

## Methods

We evaluated the cohort of 58 patients treated between 1999 and 2014 previously described by Van Leeuwen et al. [17]. All patients had a histologically confirmed locally advanced cervical carcinoma and were treated with curative intent using radiotherapy plus hyperthermia. Radiotherapy consisted of external beam irradiation (23–28 fractions of 1.8–2 Gy), which was combined with 4–5 weekly hyperthermia sessions applied shortly after a radiotherapy fraction. This schedule was followed by a 24-Gy pulsed dose rate brachytherapy boost.

**Fig. 1:**
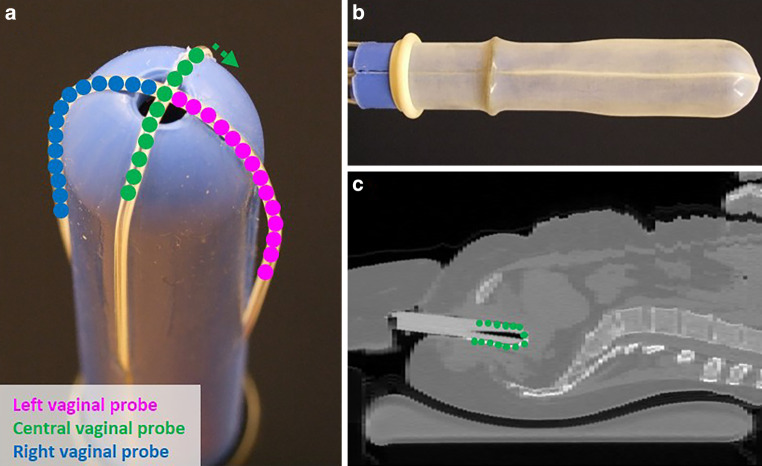
**a** Silicone elastomer mold applicator (diameter 25 mm) used to fixate intravaginal thermometry probes to measure target temperatures during hyperthermia treatments of cervical cancer patients. Three 14-sensor thermocouple probes (*left*, *central*, *right*) with 5‑mm sensor spacing are positioned along the grooves in the mold applicator. **b** The mold applicator and the thermocouple probes are covered with a condom before insertion into the patient’s vagina. **c** Hyperthermia planning CT scan in hyperthermia treatment position with thermometry in situ

Hyperthermia was applied using the 70 MHz AMC‑4 phased array system [32], consisting of four waveguides with individual phase amplitude control to focus heating to the target location. Target temperatures were measured every 30 s using three 14-sensor thermocouple probes (0.5-cm spacing) mounted on a silicone elastomer mold applicator that fixates the thermometry probes in the vaginal cavity (Fig. 1). These temperatures at the apex of the vaginal cylinder are considered representative for the temperatures achieved in the tumor region, since locoregional hyperthermia yields a focal heating zone measuring a quarter of the wavelength in tissue, which is about 10–15 cm at 70 MHz. Additional thermometry probes for locoregional treatment control were positioned in the bladder and rectum. The steady-state period started when 41 °C was registered in the target, or after a 30-min induction period, whichever was shortest; steady-state was aimed for 60 min. Phase amplitude settings were optimized at the start of treatment and adjusted during treatment, if necessary, to avoid hotspot complaints, retaining tumor heating at the same level whenever possible [14, 15].

Treatment quality per session was reported by indexed temperatures T10, T50, and T90, i.e., the temperatures at least achieved in 10, 50, and 90% of the measurement points during the steady-state period, respectively [11, 12]. These indexed temperatures represent a temperature–volume histogram, similar to dose–volume histograms in radiotherapy.

### Biological modelling

Biological modelling was performed based on the calculation model of the in-house developed software package X‑Term [25]. These calculations use an extended LQ model, with radiation sensitivity parameters α and β depending on the local temperature and the time interval between radiotherapy and hyperthermia.

#### Tumor tissue

In tumor cells, hyperthermia induces radiosensitization but also direct cytotoxicity, which is accounted for by an Arrhenius relationship. The Arrhenius parameters and temperature-dependent α and β parameters for cervical cancer were derived from extensive preclinical experiments [33]. The equivalent radiation dose EQD_RT_ is then calculated as

1 $$\mathrm{EQ}D_{\mathrm{RT}}\left(T{,}t_{\mathrm{int}}{,}D{,}d_{\mathrm{ref}}\right)=\frac{\alpha \left(T{,}t_{\mathrm{int}}\right)\cdot D+G\cdot \beta \left(T{,}t_{\mathrm{int}}\right)\cdot D^{2}}{\alpha _{37}+\beta _{37}\cdot d_{\mathrm{ref}}}+D_{\text{direct}\,\mathrm{cell}\mathrm{kill}}\left(T\right)$$

with α_37_ (Gy^−1^) and β_37_ (Gy^−2^) the normothermic LQ parameters, and α(*T,t*_*int*_) and β(*T,t*_*int*_) the LQ parameters with hyperthermia at temperature *T* and a time interval between radiotherapy and hyperthermia *t*_*int*_ (h). The preclinical data used for this model indicate that values α(*T,t*_*int*_) and β(*T,t*_*int*_) are symmetric around *t*_*int*_ = 0, which means that the effect of radiosensitization does not depend on the treatment order (i.e., hyperthermia before or after radiotherapy) [33]. *G* is the Lea–Catcheside protraction factor from the generalized LQ model, *D* (Gy) the total (physical) radiation dose, and *d*_*ref*_ (Gy) the fraction size of the reference treatment, i.e., 1.8 or 2 Gy [25]. Direct hyperthermic cytotoxicity is reflected by the term *D*_*direct cell kill*_(*T*)*,* as given by

2 $$D_{\text{direct}\,\mathrm{cell}\mathrm{kill}}\left(T\right)=\frac{7.38\cdot 10^{13}\cdot \left(T+273.15\right)\cdot exp\left(\frac{\Updelta S}{2}-\frac{\Updelta H}{2\cdot \left(T+273.15\right)}\right)}{\alpha _{37}+\beta _{37}\cdot d_{\mathrm{ref}}}$$

with ∆S = 392.08 cal/°C/mol and ∆H = 147,908.8 cal/mol [33].

#### Normal tissue

Accurate temperature-dependent LQ parameters for normal tissue are lacking, but literature data suggest a level of radiosensitization similar to tumors for simultaneous thermoradiotherapy [18]. However, the decay of radiosensitization with increasing time interval appears faster in normal tissue. Therefore, we assumed similar temperature-dependent enhancement factors of α and β as for tumor tissue, but with a faster decay rate, based on an exponential fit to the data published by Overgaard [18]. For the ratio α_37_/β_37_ a conventional value of 3 Gy was assumed [34]. Since normal tissue is not hypoxic, direct cell kill at clinical temperature levels was considered negligible.

### Equivalent dose calculation for a patient cohort

For each hyperthermia session the time interval *t*_*int*_ was determined, defined as the time between the end of the radiotherapy fraction (beam off) and the start of the steady-state hyperthermia period. The registered T50 temperature and *t*_*int*_ were used to calculate the enhancement in EQD_RT_ D50 for the radiotherapy fractions combined with hyperthermia using Eq. 1. This enhancement was summed for the 4–5 hyperthermia sessions applied, yielding the total dose enhancement over the treatment course for each patient. Although D90 would be more relevant in the radiation dose evaluation, D50 was chosen because T50 is a more robust hyperthermia evaluation parameter than T90. The thermal dose parameter TRISE (i.e. the T50 temperature rise above 37 °C, multiplied with the duration of the session for all sessions, normalised to the scheduled total treatment time) is based on T50 and was clinically shown to correlate significantly with local control in cervical cancer patients treated with thermoradiotherapy [8]. Note that the modelled hyperthermia effect yields an enhanced EQD_RT_ only for the 4–5 fractions that are directly combined with hyperthermia. For the remaining radiotherapy fractions the enhancement in EQD_RT_ is considered negligible and EQD_RT_ is the standard fraction dose of 1.8 or 2 Gy. Furthermore, since there is a time gap of several days between the external beam irradiation and the brachytherapy boost, hyperthermia-induced enhancement of the brachytherapy dose was considered negligible.

#### The influence of logistics

The time interval can vary substantially between different patients for logistic reasons. In our previous study, mean time intervals varied roughly between 30 min and 2 h [17]. All patients received the complete treatment in our hospital, but when patients receive radiotherapy elsewhere and need to travel for the hyperthermia, time intervals even up to 4 h could occur [35]. We therefore also evaluated the influence of both very optimal (*t*_*int*_ = 30 min) and suboptimal (*t*_*int*_ = 4 h) logistics on treatment effect in terms of EQD_RT_ enhancement for the complete patient cohort.

### Individual treatment evaluation using biological modelling

To illustrate the possible use of biological treatment evaluation for individual patients in daily clinical practice, two hyperthermia sessions of different patients were analyzed in detail, evaluating heterogeneity along the thermometry probes, and differentiating between tumor and normal tissue. The achieved hyperthermia temperatures, time interval, and fraction dose were used to calculate an equivalent dose–volume histogram for the specific radiotherapy fraction, with a 95% confidence band reflecting the uncertainty in α and β parameters [33]. Also here, EQD_RT_ was evaluated both for registered time intervals and for 30 min and 4 h.

## Results

### Equivalent dose calculation for the patient cohort

**Fig. 2:**
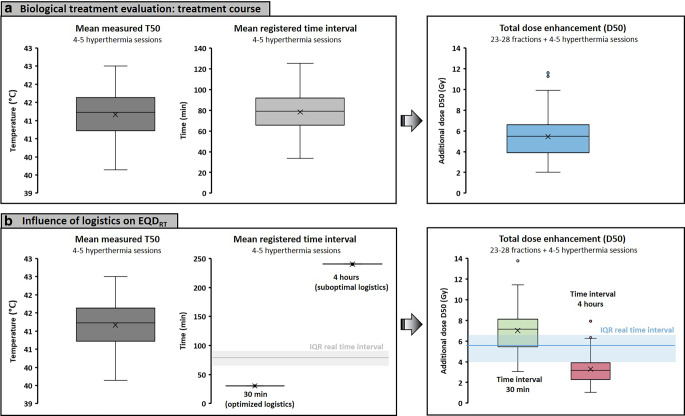
**a** Clinical measurement data (target temperatures and time intervals) registered for 58 patients with locally advanced cervical cancer treated with radiotherapy plus hyperthermia. These data were used as input for biological treatment evaluation to determine the effective total dose enhancement over the full treatment course. **b** Evaluation of the expected impact of changes in treatment logistics on the dose enhancement. A 30-min time interval reflects optimized logistics, while a 4‑h time interval represents worst-case suboptimal logistics (e.g., in case of long travelling distances). The *shaded areas* correspond to the interquartile ranges (IQR) for the real time intervals from **a** for easy comparison. *EQD*_*RT*_ Equivalent radiation dose. *T50* The temperatures at least achieved in 50% of the measurement points during the steady-state period

The boxplots in Fig. 2a summarize the average measured T50 and registered time intervals for the cohort of 58 patients. The median average measured T50 and time interval were 41.2 °C (range 39.7–42.5 °C) and 79 min (range 34–125 min), respectively. Calculation of the dose enhancement for all treatment sessions resulted in a median total enhancement in equivalent D50 of 5.5 Gy (interquartile range [IQR] 4.0–6.6 Gy) to the physical 46–50.4 Gy radiation dose.

Figure 2b shows the expected impact of very optimal logistics (*t*_*int*_ = 30 min) and suboptimal logistics (*t*_*int*_ = 4 h) compared to the real time intervals. A time interval of 30 min for all treatment sessions would significantly increase the D50 dose enhancement by ~30% to 7.1 Gy (IQR 5.5–8.1 Gy; *p* < 0.001). In case of 4‑h time intervals, an ~ 40% decrease in dose enhancement could be expected: 3.2 Gy (IQR 2.3–3.8 Gy; *p* < 0.001).

### Individual treatment evaluation using biological modelling

More detailed biological treatment evaluation can be performed for individual treatment sessions, to provide more insight in dose heterogeneity and treatment-related dose–volume histograms. To illustrate this, two treatment sessions of different patients are evaluated.

**Fig. 3:**
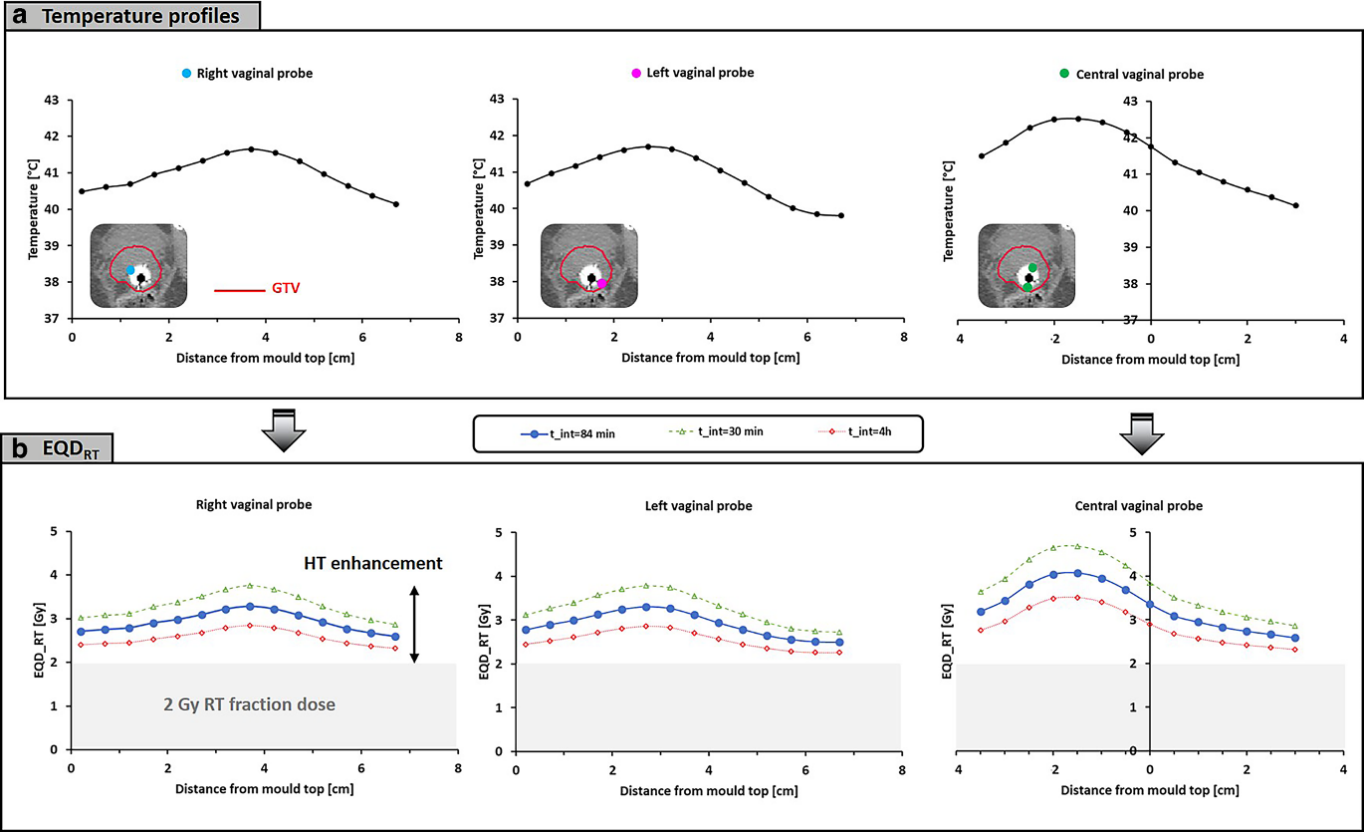
**a** Clinical temperature profiles per target probe during a hyperthermia session for patient 1, averaged over the 1‑h steady-state period. **b** Equivalent fraction dose (EQD_RT_) calculated based on the measured temperatures and the registered time interval of 86 min (*blue curves*). The *grey* region represents the 2‑Gy fraction dose, so the enhancement by hyperthermia for this fraction is the dose beyond 2 Gy. The *green* and *red dashed* *curves* represent the equivalent dose estimates if the time interval were to have been 30 min or 4 h, respectively

*Patient 1* is a 76-year-old woman with a locally advanced cervical tumor FIGO (Federation Internationale de Gynecologie et d’Obstetrique) stage 4A, who received 23 × 2 Gy plus five sessions of hyperthermia followed by brachytherapy. The time interval between the radiotherapy fraction and this hyperthermia session was 86 min. Figure 3a shows the measured temperatures along the target thermometry probes, averaged over the 60 min steady-state period. As shown on the CT scan, the GTV delineation fully encloses the thermometry probes, so all measurements indicate target temperatures. Overall, the registered average temperatures varied between 39.8 and 42.5 °C because of local variations in power deposition and perfusion inherent to locoregional hyperthermia. Figure 3b shows the corresponding EQD_RT_ for each measurement point, which predicts an overall increase from 2 Gy to 2.50–4.07 Gy, depending on the location. If logistics were optimized to realize a 30-min time interval, the dose range would become 2.68–4.73 Gy. For a 4‑h time interval, this would reduce to 2.26–3.51 Gy.

**Fig. 4:**
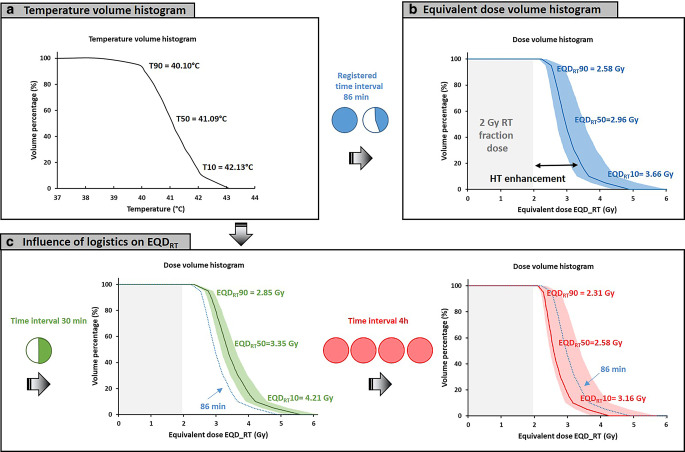
**a** Temperature–volume histogram for a hyperthermia session of patient 1 based on continuous measurements during the 1‑h steady-state period. **b** Equivalent dose–volume histogram with 95% confidence band based on the temperature–volume histogram and registered time interval of 86 min. **c** The expected influence of logistics on the equivalent dose–volume histogram. For easy comparison the *solid blue line* from **b** was redrawn here as a *dotted line*

Figure 4a summarizes the treatment session in a temperature–volume histogram over the steady-state period, with indexed temperatures T90, T50, and T10 of 40.1, 41.1, and 42.1 °C, respectively. Figure 4b shows the corresponding equivalent dose–volume histogram, with an equivalent D50 fraction dose of 2.96 Gy (95% CI: 2.70–3.54 Gy). Figure 4c shows equivalent dose–volume histograms for optimal (*t*_*int*_ = 30 min) and suboptimal (*t*_*int*_ = 4 h) logistics, yielding an equivalent D50 of 3.35 Gy (95% CI: 3.15–3.60 Gy) and 2.58 Gy (95% CI: 2.47–3.38 Gy), respectively. With increasing time interval, the dose–volume histogram becomes steeper, i.e., the difference between D90 and D10 decreases, though also the overall effective enhancement reduces.

**Fig. 5:**
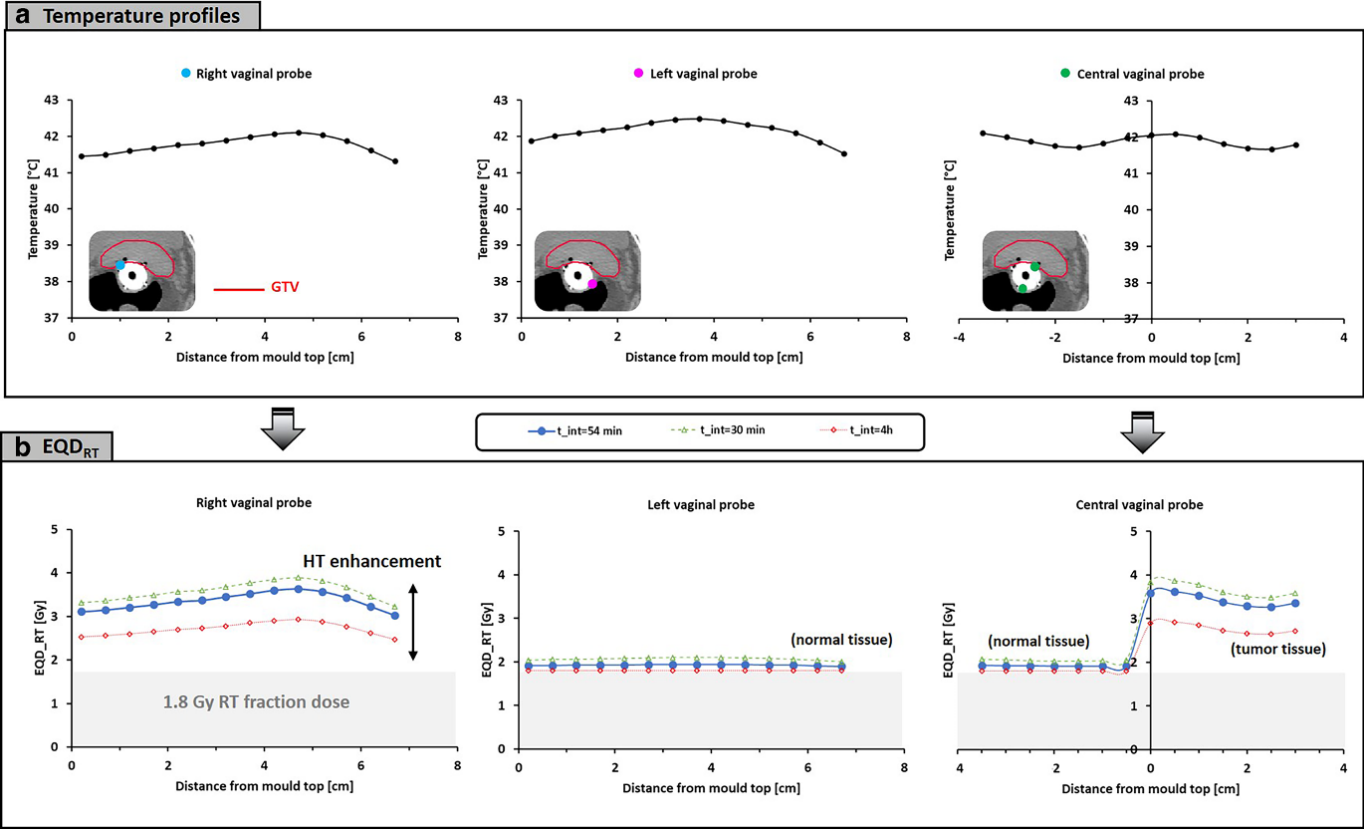
**a** Clinical temperature profiles per target probe during a hyperthermia session for patient 2, averaged over the 1‑h steady-state period. **b** Equivalent fraction dose (EQD_RT_) calculated based on the measured temperatures and the registered time interval of 54 min (*blue curves*). The *green* and *red dashed curves* represent the equivalent fraction dose estimates if the time interval were to have been 30 min or 4 h, respectively. The *grey* region represents the 1.8 Gy fraction dose, so the enhancement by hyperthermia for this fraction is the dose beyond 1.8 Gy. Note that the left vaginal probe and half of the central vaginal probe sensors represent normal tissue

*Patient 2* is a 72-year-old woman with a locally advanced cervical tumor FIGO stage 3B and paraaortic lymph node involvement, who received a 28 × 1.8 Gy plus five sessions of hyperthermia followed by brachytherapy. The time interval between the radiotherapy fraction and this hyperthermia session was 54 min. Figure 5a shows the measured temperatures along the target thermometry probes averaged over the 60 min steady-state period. As shown on the CT scan, the GTV temperatures are only represented by the right vaginal probe and half of the central vaginal probe; other measurements are labelled normal tissue. Overall, measured tumor temperatures varied between 41.3 and 42.1 °C; normal tissue temperatures varied between 41.5 and 42.5 °C. Figure 5b shows the corresponding EQD_RT_ for each measurement point, which predicts an overall increase from 1.8 Gy to 3.03–3.63 Gy in tumor dose, while the normal tissue dose is enhanced minimally to 1.9–1.94 Gy. If logistics were to be optimized to realize a 30-min time interval, the GTV dose range would become 3.23–3.89 Gy; for a 4‑h time interval this would reduce to 2.47–2.93 Gy. Normal tissue enhancement is practically absent with longer time intervals and varies only mildly up to 0.3 Gy for optimized logistics, illustrating the tumor selectiveness of hyperthermic radiosensitization.

**Fig. 6:**
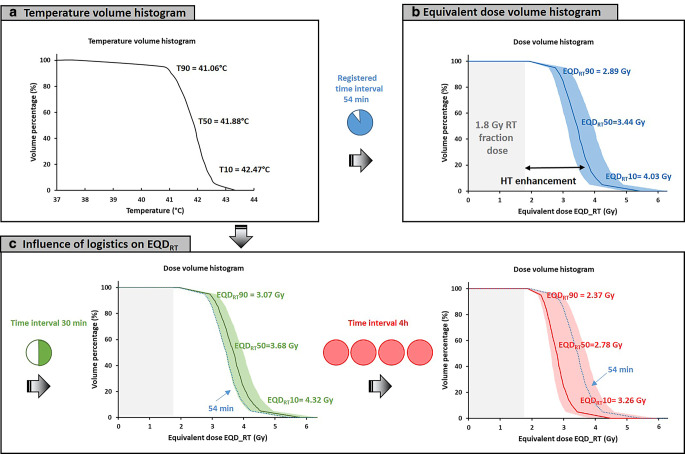
**a** Temperature–volume histogram for a hyperthermia session of patient 2 based on continuous measurements during the 1‑h steady-state period. **b** Equivalent dose–volume histogram with 95% confidence band based on the temperature–volume histogram and registered time interval of 54 min. **c** The expected influence of logistics on the equivalent dose–volume histogram. For easy comparison, the *solid blue line* from **b** was redrawn here as a *dotted line*

Figure 6a summarizes the session in a temperature–volume histogram over the steady-state period, with indexed temperatures T90, T50, and T10 of 41.1, 41.9, and 42.5 °C, respectively. Figure 6b shows the corresponding equivalent dose–volume histogram, with an equivalent D50 fraction dose of 3.44 Gy (95% CI: 3.12–3.96 Gy). Figure 6c shows equivalent dose–volume histograms for optimal (*t*_*int*_ = 30 min) and suboptimal (*t*_*int*_ = 4 h) logistics. The observed impact of logistics on dose enhancement is somewhat larger compared to patient 1, because of the higher temperatures achieved. The predicted equivalent D50 is 3.68 Gy (95% CI: 3.42–3.99 Gy) and 2.78 Gy (95% CI: 2.59–3.72 Gy), for a 30-min and 4‑h time interval, respectively.

## Discussion

This study demonstrated clinical use of biological modelling for treatment evaluation in thermoradiotherapy by calculating the enhanced equivalent radiation dose (EQD_RT_) based on clinically measured temperatures and recorded time intervals between radiotherapy and hyperthermia. It was applied to locally advanced cervical cancer patients unfit for chemotherapy and therefore treated with thermoradiotherapy instead of standard chemoradiation. This is a standard indication for thermo-radiotherapy in the Netherlands because of equal effectiveness [5]. Results showed a median total dose enhancement (D50) of 5.5 Gy by hyperthermia over all patients; enhancement for individual patients depends strongly on the achieved temperature levels and time intervals.

The widely accepted optimal goal temperature for hyperthermia is 43 °C [11], and aiming for high temperature levels is important because of the clinical thermal dose–effect relationship [8, 9]. However, achieved temperatures are usually limited by treatment-limiting hotspots in normal tissue, despite effective treatment planning and/or adequate steering protocols. Since further increasing the tumor temperatures is challenging, optimizing logistics to realize a workflow enabling short time intervals of about 30 min is a relatively easy and effective method to further optimize treatment efficacy. As results of this paper indicated, optimizing logistics could increase the dose enhancement by another ~30% at the same temperature levels and without significant enhancement in normal tissue. Application of twice weekly hyperthermia is another conceivable strategy to further increase EQD_RT_. However, development of thermotolerance is likely to significantly limit the effect of extra hyperthermia treatments [36]. A clinical study comparing hyperthermia once or twice per week for superficial tumors showed no significant difference [37].

The reduced effectiveness of hyperthermia for longer time intervals is logical since an important working mechanism of hyperthermia is DNA damage repair inhibition and most of the repair takes place within 2 h [17, 38]. Although the effectiveness of hyperthermia decreases for longer time intervals, hyperthermia has multiple working mechanisms and beneficial effects remain up to several hours [35]. For example, reoxygenation may last up to 24 h [39, 40] and direct cytotoxic effects on hypoxic cells are intrinsically independent of the time interval. Up to now, the biological models used include DNA damage repair inhibition and direct cytotoxicity. This means that the EQD_RT_ calculation currently does not account for other relevant hyperthermia mechanisms, such as reoxygenation and immunologic effects, which are also important. This also explains the fact that the modelled hyperthermic enhancement only influences the EQD_RT_ of the radiotherapy fractions directly combined with the weekly hyperthermia sessions. Inclusion of other hyperthermia working mechanisms into the model would yield a stronger enhancement, and part of the enhancement could extend for more than one radiotherapy fraction, e.g., in case of reoxygenation or immune response. Therefore, the EQD_RT_ calculated in this paper is an underestimation of the real equivalent dose achieved in thermoradiotherapy treatments and including these other mechanisms would likely yield some additional Gy in EQD_RT_.

The challenge of real dose predictions is general in biological modelling and not specific for thermoradiotherapy modelling. For radiotherapy alone, uncertainties in LQ parameter values also influence exact dose predictions [41]. Biological modelling can also be applied to chemoradiation [42], and a study by Plataniotis and Dale predicted a chemotherapy equivalent dose enhancement of only 1.2–2.6 Gy for tumors with intermediate radiosensitivity, which could increase up to 8 Gy best case for tumors with very low radiosensitivity [43]. This chemotherapeutic enhancement is relatively low and quite likely also an underestimation, given the equal effectiveness of chemoradiation and thermoradiation suggested in the RADCHOC study [5], and the underestimation by our biological model for thermoradiation not yet including all relevant hyperthermia mechanisms.

Nevertheless, despite general uncertainties in biological modelling, it is quite useful for qualitative evaluations and biological treatment evaluation for thermoradiotherapy, since in its present form it still gives a qualitative indication of achieved treatment quality in addition to temperature only. The biological treatment evaluation provides insight into the effect of time interval, which is not accounted for in standard thermal dose evaluation and allows qualitative evaluation of dose heterogeneity and different treatment scenarios with varying time intervals. Future research aims at more advanced models also including other relevant hyperthermia mechanisms [19]. This challenging task requires carefully designed preclinical experiments to obtain essential modelling parameters as a function of temperature and time interval.

At our department, hyperthermia is applied after radiotherapy, and based on results of a previous retrospective analysis suggesting that a short time interval improves overall survival [17], we aimed to optimize logistics to shorten time intervals. To this end, the radiotherapy treatment schedule was coordinated such that on hyperthermia treatment days, the radiotherapy session finishes close to the start of the scheduled hyperthermia session. Furthermore, we became more reluctant to allow patients receiving radiotherapy treatments in other hospitals and travel for hyperthermia.

When hyperthermia is applied after radiotherapy, achieving a time interval of about 30 min is quite optimal, especially since the warmup time, i.e., the time required before steady-state temperatures are reached, is included in that time interval. Previous research has shown that the enhancement decreases strongly with increasing time interval, especially at relatively high temperatures, and that most of the enhancement is already lost after 1 h [27]. This implies that even better results could be obtained with time intervals shorter than 30 min. This could be realized when hyperthermia is applied first. Such advanced logistics were successfully implemented by Notter et al. for treatment of large-sized (heavily pretreated) recurrent breast cancer with hypofractionated reirradiation (4 Gy once a week, up to 20 Gy) and hyperthermia [44]. Patients received a 45–60-min hyperthermia treatment using water-filtered infrared A (wIRA) and treatment stopped when the linear accelerator became available. During transfer to radiotherapy, patients were thermally shielded with a preheated blanket. This protocol was applied to 73 patients, resulting in a 59% local control rate throughout their lifetime after complete response of macroscopic disease. The biological model applied in the present paper can also be used to predict radiosensitization for this reversed sequence. As remarked in the methods, the values α(*T,t*_*int*_) and β(*T,t*_*int*_) are based on preclinical data, which include the effects of direct cell kill and inhibition of DNA repair. These effects show symmetry around *t*_*int*_ = 0, and the modelled effect of radiosensitization therefore does not depend on the treatment order, i.e., hyperthermia before or after radiotherapy. Inclusion of other effects like reoxygenation and immunologic effects will result in more effective radiosensitization, as discussed above, which then potentially become sequence dependent.

The temperature heterogeneity also yields substantial heterogeneities in the equivalent dose distribution, as observed when evaluating EQD_RT_ along the thermometry probes for individual patients. Homogeneous temperatures are difficult to achieve during locoregional hyperthermia due to heterogeneities in dielectric and thermal tissue properties and perfusion, combined with limited steering possibilities of the power deposition caused by the large wavelength (~50 cm in muscle tissue) at frequencies around 100 MHz. Future efforts to improve homogeneity of the EQD_RT_ will therefore focus on combined (robust) optimization strategies, adjusting radiation dose prescription by optimization based on EQD_RT_ instead of physical dose. A novel planning tool for radiobiological optimization of thermoradiotherapy has recently been implemented in RayStation (RaySearch Laboratories AB, Stockholm, Sweden) [45]. Optimization based on EQD_RT_ enables the use of standard dose constraints and objectives, which allows relatively easy interpretation of the combined treatment plan. Research on robust optimization strategies is ongoing, accounting also for uncertainties in hyperthermia delivery [46].

Overall, biological modelling provides comprehensible dose parameter estimates that improve insight into the overall treatment quality in addition to standard thermal (dose) parameters. These insights can eventually also help to optimize treatment schedules and standardize clinical protocols among hyperthermia centers.

## Conclusion

Biological treatment evaluation is a useful addition to standard thermal (dose) evaluation of hyperthermia treatments. The current model accounts for a subset of relevant hyperthermia effects, i.e., direct hyperthermic cytotoxicity and DNA damage repair inhibition. This implies that current equivalent dose predictions underestimate the real effect, which is also enhanced by other relevant mechanisms such as reoxygenation and immunologic effects. Including these mechanisms in an accurate fashion in biological models is challenging and subject to further research. Nevertheless, the current biological model still provides relevant qualitative insight into differences in treatment quality between different treatment strategies in terms of expected radiation dose enhancement, also accounting for the influence of the time interval between both treatment modalities. When evaluating our cervical cancer patient cohort of 58 patients, we found a median dose enhancement (D50) of 5.5 Gy over the full treatment course. Optimization of logistics aiming for shorter but feasible 30-min time intervals seems worthwhile to maximize treatment efficacy, with an expected increase of ~30% in total dose enhancement.

## References

[1] Sung H, Ferlay J, Siegel RL, Laversanne M, Soerjomataram I, Jemal A, Bray F (2021). Global cancer statistics 2020: GLOBOCAN estimates of incidence and mortality worldwide for 36 cancers in 185 countries. CA Cancer J Clin.

[2] Chemoradiotherapy for Cervical Cancer Meta-Analysis Collaboration (2008). Reducing uncertainties about the effects of chemoradiotherapy for cervical cancer: a systematic review and meta-analysis of individual patient data from 18 randomized trials. J Clin Oncol.

[3] Kok HP, Cressman ENK, Ceelen W, Brace CL, Ivkov R, Grull H, Ter Haar G, Wust P, Crezee J (2020). Heating technology for malignant tumors: a review. Int J Hyperthermia.

[4] van der Zee J, González D, van Rhoon GC, van Dijk JDP, van Putten WLJ, Hart AA (2000). Comparison of radiotherapy alone with radiotherapy plus hyperthermia in locally advanced pelvic tumours: a prospective, randomised, multicentre trial. Dutch deep hyperthermia group. Lancet.

[5] Lutgens LC, Koper PC, Jobsen JJ, van der Steen-Banasik EM, Creutzberg CL, van den Berg HA, Ottevanger PB, van Rhoon GC, van Doorn HC, Houben R, van der Zee J (2016). Radiation therapy combined with hyperthermia versus cisplatin for locally advanced cervical cancer: results of the randomized RADCHOC trial. Radiother Oncol.

[6] Datta NR, Stutz E, Gomez S, Bodis S (2019). Efficacy and safety evaluation of the various therapeutic options in locally advanced cervix cancer: a systematic review and network meta-analysis of randomized clinical trials. Int J Radiat Oncol Biol Phys.

[7] Franckena M, Stalpers LJ, Koper PC, Wiggenraad RG, Hoogenraad WJ, van Dijk JD, Warlam-Rodenhuis CC, Jobsen JJ, van Rhoon GC, van der Zee J (2008). Long-term improvement in treatment outcome after radiotherapy and hyperthermia in locoregionally advanced cervix cancer: an update of the Dutch deep hyperthermia trial. Int. J. Radiat. Oncol. Biol. Phys..

[8] Franckena M, Fatehi D, de Bruijne M, Canters RA, van Norden Y, Mens JW, van Rhoon GC, van der Zee J (2009). Hyperthermia dose-effect relationship in 420 patients with cervical cancer treated with combined radiotherapy and hyperthermia. Eur. J. Cancer.

[9] Ohguri T, Harima Y, Imada H, Sakurai H, Ohno T, Hiraki Y, Tuji K, Tanaka M, Terashima H (2018). Relationships between thermal dose parameters and the efficacy of definitive chemoradiotherapy plus regional hyperthermia in the treatment of locally advanced cervical cancer: data from a multicentre randomised clinical trial. Int J Hyperthermia.

[10] Stoll AM, Greene LC (1959). Relationship between pain and tissue damage due to thermal radiation. J Appl Physiol.

[11] Sapareto SA, Dewey WC (1984). Thermal dose determination in cancer therapy. Int. J. Radiat. Oncol. Biol. Phys..

[12] Bruggmoser G, Bauchowitz S, Canters R, Crezee H, Ehmann M, Gellermann J, Lamprecht U, Lomax N, Messmer MB, Ott O, Abdel-Rahman S, Schmidt M, Sauer R, Thomsen A, Wessalowski R, van Rhoon G, Atzelsberg Research Group, European Society for Hyperthermic Oncology (2012). Guideline for the clinical application, documentation and analysis of clinical studies for regional deep hyperthermia: quality management in regional deep hyperthermia. Strahlenther Onkol.

[13] van Rhoon GC (2016). Is CEM43 still a relevant thermal dose parameter for hyperthermia treatment monitoring?. Int J Hyperthermia.

[14] Kok HP, Korshuize-van Straten L, Bakker A, de Kroon-Oldenhof R, Geijsen ED, Stalpers LJA, Crezee J (2017). On-line adaptive hyperthermia treatment planning during locoregional heating to suppress treatment limiting hot spots. Int J Radiat Oncol Biol Phys.

[15] Kok HP, Crezee J (2022). Adapt2Heat: treatment planning-assisted locoregional hyperthermia by on-line visualization, optimization and re-optimization of SAR and temperature distributions. Int J Hyperthermia.

[16] Canters RA, Franckena M, van der Zee J, Van Rhoon GC (2008). Complaint-adaptive power density optimization as a tool for HTP-guided steering in deep hyperthermia treatment of pelvic tumors. Phys. Med. Biol..

[17] van Leeuwen CM, Oei AL, Chin KWTK, Crezee J, Bel A, Westermann AM, Buist MR, Franken NAP, Stalpers LJA, Kok HP (2017). A short time interval between radiotherapy and hyperthermia reduces in-field recurrence and mortality in women with advanced cervical cancer. Radiat Oncol.

[18] Overgaard J (1980). Simultaneous and sequential hyperthermia and radiation treatment of an experimental tumor and its surrounding normal tissue in vivo. Int. J. Radiat. Oncol. Biol. Phys..

[19] Kok HP, van Rhoon GC, Herrera TD, Overgaard J, Crezee J (2022). Biological modeling in thermoradiotherapy: present status and ongoing developments toward routine clinical use. Int J Hyperthermia.

[20] Sanchez-Nieto B, Nahum AE (2000). BIOPLAN: software for the biological evaluation of. Radiotherapy treatment plans. Med Dosim.

[21] Stavrev PV, Stavreva N, Ruggieri R, Nahum AE, Tsonev P, Penev D, Pressyanov D (2021). Theoretical investigation of the impact of different timing schemes in hypofractionated radiotherapy. Med Phys.

[22] Fowler JF (1989). The linear-quadratic formula and progress in fractionated radiotherapy. Br. J. Radiol..

[23] Crezee J, van Leeuwen CM, Oei AL, van Heerden LE, Bel A, Stalpers LJ, Ghadjar P, Franken NA, Kok HP (2016). Biological modelling of the radiation dose escalation effect of regional hyperthermia in cervical cancer. Radiat Oncol.

[24] Kok HP, Crezee J, Franken NAP, Stalpers LJA, Barendsen GW, Bel A (2014). Quantifying the combined effect of radiation therapy and hyperthermia in terms of equivalent dose distributions. Int. J. Radiat. Oncol. Biol. Phys..

[25] van Leeuwen CM, Crezee J, Oei AL, Franken NA, Stalpers LJ, Bel A, Kok HP (2017). 3D radiobiological evaluation of combined radiotherapy and hyperthermia treatments. Int J Hyperthermia.

[26] Myerson RJ, Roti Roti JL, Moros EG, Straube WL, Xu M (2004). Modelling heat-induced radiosensitization: clinical implications. Int. J. Hyperthermia.

[27] Kok HP, Herrera TD, Crezee J (2023). The relevance of high temperatures and short time intervals between radiation therapy and hyperthermia: insights in terms of predicted equivalent enhanced radiation dose. Int J Radiat Oncol Biol Phys.

[28] Kok HP, Van Dijk I, Crama KF, Franken NAP, Rasch CRN, Merks JHM, Crezee J, Balgobind BV, Bel A (2019). Reirradiation plus hyperthermia for recurrent pediatric sarcoma; a simulation study to investigate feasibility. Int J Oncol.

[29] van Leeuwen CM, Crezee J, Oei AL, Franken NAP, Stalpers LJA, Bel A, Kok HP (2018). The effect of time interval between radiotherapy and hyperthermia on planned equivalent radiation dose. Int J Hyperthermia.

[30] Androulakis I, Mestrom RMC, Christianen M, Kolkman-Deurloo IK, van Rhoon GC (2022). A novel framework for the optimization of simultaneous ThermoBrachytherapy. Cancers.

[31] Ghaderi Aram M, Zanoli M, Nordstrom H, Toma-Dasu I, Blomgren K, Trefna HD (2021). Radiobiological evaluation of combined gamma knife radiosurgery and hyperthermia for pediatric neuro-oncology. Cancers.

[32] van Dijk JDP, Schneider CJ, van Os RM, Blank LE, González DG (1990). Results of deep body hyperthermia with large waveguide radiators. Adv. Exp. Med. Biol..

[33] van Leeuwen CM, Oei AL, Ten CR, Franken NA, Bel A, Stalpers LJA, Crezee J, Kok HP (2018). Measurement and analysis of the impact of time-interval, temperature and radiation dose on tumour cell survival and its application in thermoradiotherapy plan evaluation. Int J Hyperthermia.

[34] Williams MV, Denekamp J, Fowler JF (1985). A review of alpha/beta ratios for experimental tumors: implications for clinical studies of altered fractionation. Int J Radiat Oncol Biol Phys.

[35] Kroesen M, Mulder HT, van Holthe JML, Aangeenbrug AA, Mens JWM, van Doorn HC, Paulides MM, Oomen-de Hoop E, Vernhout RM, Lutgens LC, van Rhoon GC, Franckena M (2019). The effect of the time interval between radiation and hyperthermia on clinical outcome in 400 locally advanced cervical carcinoma patients. Front Oncol.

[36] Overgaard J, Nielsen OS (1983). The importance of thermotolerance for the clinical treatment with hyperthermia. Radiother Oncol.

[37] Engin K, Tupchong L, Moylan DJ, Alexander GA, Waterman FM, Komarnicky L, Nerlinger RE, Leeper DB (1993). Randomized trial of one versus two adjuvant hyperthermia treatments per week in patients with superficial tumours. Int. J. Hyperthermia.

[38] Krawczyk PM, Eppink B, Essers J, Stap J, Rodermond H, Odijk H, Zelensky A, van Bree C, Stalpers LJ, Buist MR, Soullie T, Rens J, Verhagen HJ, O’Connor MJ, Franken NA, ten Hagen TL, Kanaar R, Aten JA (2011). Mild hyperthermia inhibits homologous recombination, induces BRCA 2 degradation, and sensitizes cancer cells to poly (ADP-ribose) polymerase-1 inhibition. Proc. Natl. Acad. Sci. U.S.A..

[39] Dewhirst MW, Oleson JR, Kirkpatrick J, Secomb TW (2022). Accurate three-dimensional thermal dosimetry and assessment of physiologic response are essential for optimizing thermoradiotherapy. Cancers.

[40] Jones EL, Prosnitz LR, Dewhirst MW, Marcom PK, Hardenbergh PH, Marks LB, Brizel DM, Vujaskovic Z (2004). Thermochemoradiotherapy improves oxygenation in locally advanced breast cancer. Clin Cancer Res.

[41] van Leeuwen CM, Oei AL, Crezee J, Bel A, Franken NAP, Stalpers LJA, Kok HP (2018). The alfa and beta of tumours: a review of parameters of the linear-quadratic model, derived from clinical radiotherapy studies. Radiat Oncol.

[42] Dale RG (2022). Quantification of chemotherapy contributions to chemoradiotherapy. Radiat Phys Chem.

[43] Plataniotis GA, Dale RG (2008). Use of concept of chemotherapy-equivalent biologically effective dose to provide quantitative evaluation of contribution of chemotherapy to local tumor control in chemoradiotherapy cervical cancer trials. Int. J. Radiat. Oncol. Biol. Phys..

[44] Notter M, Piazena H, Vaupel P (2016). Hypofractionated re-irradiation of large-sized recurrent breast cancer with thermography-controlled, contact-free water-filtered infra-red-A hyperthermia: a retrospective study of 73 patients. Int J Hyperthermia.

[45] Ödén J, Pavoni B, Meschini G, Enriquez P, Crezee H, Frederiksson A, Kok P, Eriksson K (2022). A novel planning tool for radiobiological optimization of thermoradiotherapy treatments. Phys Medica.

[46] Herrera TD, Ödén J, Crezee J, Lorenzo Polo A, Kok HP (2023). Robust optimization and evaluation of radiotherapy combined with hyperthermia based on equivalent enhanced radiation dose.

